# Novel Schiff Base Derived from Amino Pyrene: Synthesis, Characterization, Crystal Structure Determination, and Anticancer Applications of the Ligand and Its Metal Complexes

**DOI:** 10.3390/molecules28217352

**Published:** 2023-10-30

**Authors:** Elham S. Aazam, Maryam A. Majrashi

**Affiliations:** Department of Chemistry, Faculty of Science, King Abdulaziz University, Jeddah P.O. Box 23622, Saudi Arabia

**Keywords:** Schiff base ligand, metal complexes, cytotoxicity, MCF-7, X-ray crystallography, IR, magnetic, electronic spectra

## Abstract

In this study, we report the cytotoxicity of a newly synthesized Schiff base HL ((E)-2-ethoxy-6((pyren-1-ylimino)methyl)phenol) and its derived metal complexes (Zn(II), Cu(II), Co(II), Cr(III), and Fe(III)) along with their structural characterizations by means of elemental analysis, magnetic moment, molar conductance, IR, UV-Vis, ESR, and mass spectrometry. The single X-ray diffraction of the HL shows that it exists in the phenol-imine form in its solid state. The NMR and IR data indicate that the bidentate binding of the Schiff base ligand with the metal center occurs during complexation through the azomethine nitrogen atom and the hydroxyl group oxygen atom of the 3-ethoxy salicylaldehyde. The electronic spectra and magnetic measurements indicate that the Co(II) complex has a tetrahedral geometry and that the Cr(III) and Fe(III) complexes have a distorted octahedral geometry. The ESR and electronic spectra suggest that the Cu(II) complex has a distorted tetrahedral geometry. The cytotoxic effects of the HL and all of the metal complexes were studied using human breast cancer (MCF-7) cells. The Cu(II) and Zn(II) complexes exhibited the highest activity against the tested cell line, with IC_50_ values of 5.66 and 12.74 μg/mL, respectively, and their activity was higher than that of the fluorouracil cancer drug against the MCF-7 cells (18.05 μg/mL).

## 1. Introduction

Breast cancer is a pressing issue for public health worldwide, being the second deadliest cancer for women. In recent years, there has been a drastic increase in breast cancer incidence. The need for the development of improved drugs for breast cancer treatment has become increasingly urgent due to the emergence of resistance in breast cancer cells following initially effective chemotherapy. To better understand breast cancer susceptibility and cellular responses to apoptosis, the estrogen receptor-positive MCF-7 cell line, derived from a patient with metastatic breast cancer, has served as a prominent model system [1].

Schiff bases, which are known for their coordination ability, hold significant importance as ligands in inorganic chemistry [2]. These compounds are synthesized through condensation reactions between amines and aldehydes or ketones under specific conditions. Schiff bases have garnered extensive research attention due to their facile formation, remarkable structural properties, strong binding capabilities, their use in diverse applications in the pharmaceutical industry, and their biological activities, such as their antibacterial [3], antifungal, and antitumor activities [4]. Notably, they play a crucial role in coordination chemistry by forming stable complexes with transition, non-transition, and inner transition metals, and their properties are significantly enhanced by doing so. Their ability to form stable complexes with metal ions is attributed to the presence of the azomethine (N=CH) lone pair of electrons in their structure. These complexes and their biological activities have been widely studied in the literature due to their selectivity towards a variety of organisms [5,6].

Heterocyclic Schiff bases are fundamentally important because of their elevated biological activities and their role in the development of novel materials with unique properties that can be modified by varying the substituents. One very interesting and promising class of heterocyclic Schiff base is the series of pyrene-based Schiff bases, which have been reported to be selective chemosensors for the detection of Fe(III) [7,8], Cu(II) [7,9], sulfide ions [9], and Al^3+^ and Ga^3+^ [10]. Srinivasan et al. developed two novel Schiff base compounds that are pyrene based, referred to as KSB-1 and KSB-2. They also investigated their antimicrobial activities against some pathogenic strains and reported that their AIE (aggregation-induced emission) properties make them prospective biomaterials for in vitro bioimaging and BSA-binding studies [8]. Recently, a monomeric Schiff base was synthesized via a reaction between o-vanillin and 1-aminopyrene. Interestingly, it exhibited a distinctive sensitivity to Sn^2+^ in comparison with various other metal ions [11]. However, the cytotoxic activities of pyrene-based Schiff base complexes are scarce or lacking.

Trace elements such as iron, copper, and zinc, along with ultra-trace elements like cobalt and chromium, are essential for supporting the proper function of various physiological and metabolic processes within living tissues. These complexes are crucial in maintaining the integrity of these processes [12].

In continuation of our work in the area of Schiff base complexes [13,14], this research paper reports the synthesis, characterization, and crystal structure of a Schiff base HL and its Zn(II), Cu(II), Co(II), Fe(III), and Cr(III) complexes. The Schiff base possesses N and O donor sites and exhibits excellent coordination potential for binding with various metal ions. Hence, it was of interest to prepare a pyrene-based Schiff base and its metal complexes to study their chemistry and evaluate their cytotoxic effects on a human breast cancer cell line (MCF-7 cells) by applying the MTT colorimetric assay.

## 2. Results and Discussion

### 2.1. Chemistry and Characterizations

**Scheme 1:**
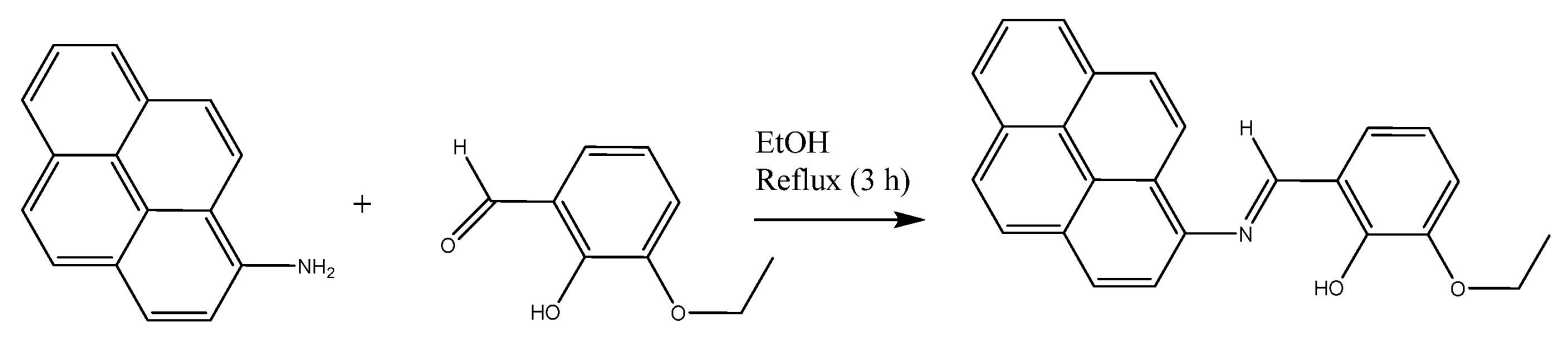
Synthesis pathway of HL.

**Figure 1:**
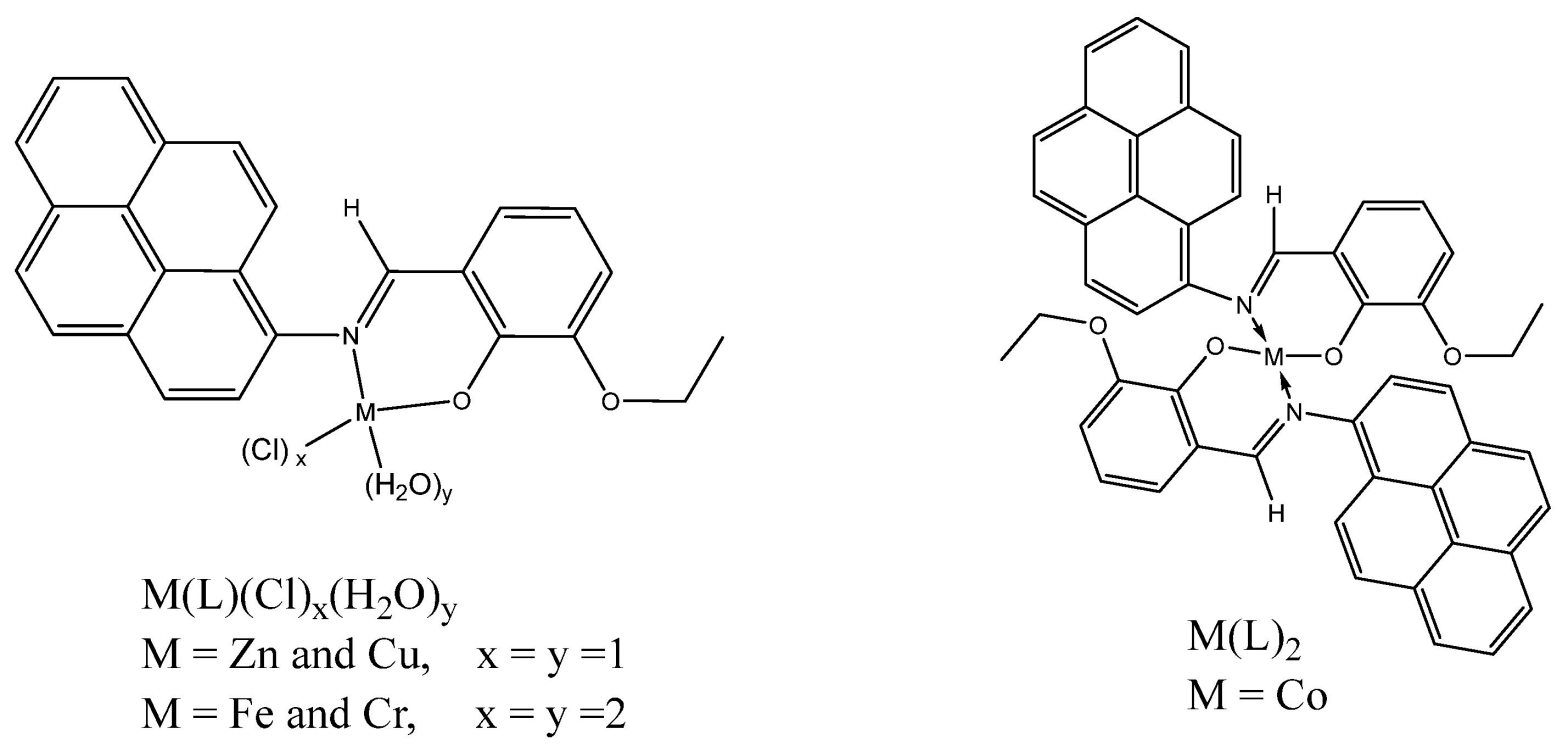
Proposed structures for metal complexes.

The HL, ((E)-2-ethoxy-6((pyren-1-ylimino)methyl)phenol), derived from 3-ethoxy salicylaldehyde and 1-aminopyrene, was prepared according to Scheme 1, and a good yield was obtained under reflux conditions using ethanol as a solvent. The products were re-crystallized via the slow evaporation of the solvent at room temperature. All new metal complexes (M(L)(Cl)_x_(H_2_O)_y_ where M = Zn or Cu, x = y = 1; M = Fe or Cr, x = y = 2; and M(L)_2_ where M = Co) were synthesized via direct reactions between the ligand and the corresponding metal chloride salts (Figure 1). The ligand and the complexes were stable at room temperature and insoluble in methanol and ethanol but soluble in DMF and DMSO. They were thoroughly characterized using FT-IR, elemental analysis, UV–Vis, ^1^H NMR, ^13^C NMR, and mass spectral studies. The structure of the Schiff base was established using X-ray crystallography. The results reveal that the Zn(II), Cu(II), and Co(II) complexes have a tetrahedral geometry, while the Fe(III) and Cr(III) complexes exhibit octahedral coordination around their central metal ions. The molar conductances of these compounds were measured in DMSO (1 × 10^−3^ M) at 25 °C, and the results indicate that they are non-electrolytic in nature (2.05–15.15 Ω^−1^ cm^2^ mol^−1^) [15]. Non-electrolytic complexes indicate that the anions are found in the coordination sphere. Also, the melting point values are greater than 210 °C. The data from the elemental analysis of the Schiff base and its complexes (Table 1) agree with the structure of the ligand shown in Scheme 1 and with the structures of the complexes shown in Figure 1.

#### 2.1.1. XRD Analysis of HL

Structure elucidation was carried out on HL using the single-crystal X-ray diffraction technique. A study of the (E)-2-ethoxy-6-((pyren-1-ylimino)methyl)phenol compound was carried out, and the data obtained are presented in Table 2. Selected geometric parameters like bond length and bond angle are listed in Table 3.

**Figure 2:**
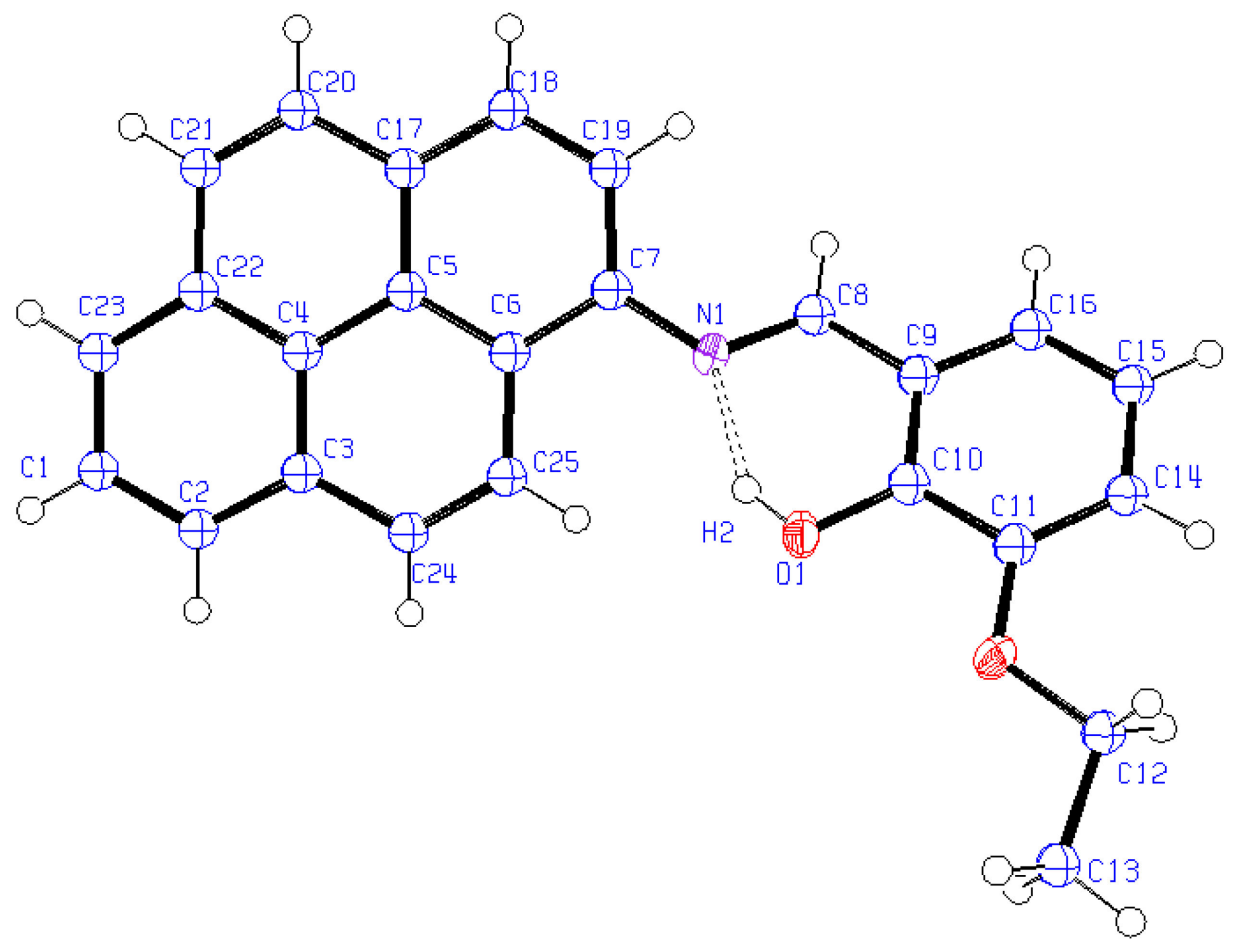
The molecular structure of HL, with atom labeling. Displacement ellipsoids are drawn at the 50% probability level.

**Figure 3:**
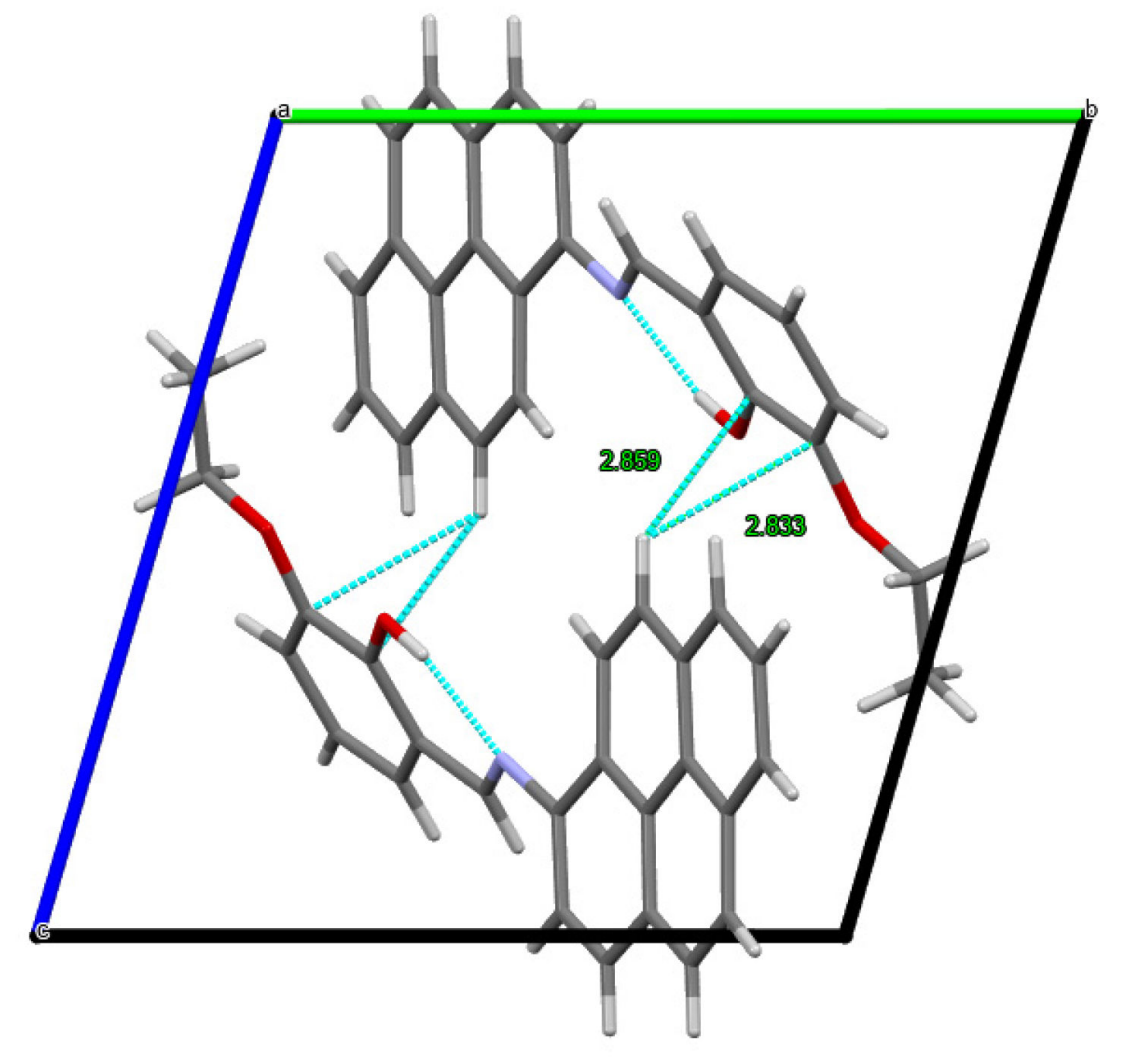
The crystal packing of HL viewed along an axis. The C-H···π interactions linking the molecules are shown as dashed lines.

The compound’s crystal structure was determined in a triclinic space group P1, with a formula unit of “C_25_H_19_NO_2_” and Z = 2. A perspective view of the compound is provided in Figure 2, which shows an intramolecular (O2-H2…N1) hydrogen bond (O…N = 2.580(3), O-H = 0.84, H…N = 1.84, and OH…N = 146.3°), involving the O-H group and the azomethine N atom, which is a characteristic hydrogen bond for Schiff bases that leads to the formation of an S(6) six-membered ring [16]. In Schiff bases, two distinct forms of intra-molecular hydrogen bonds can occur, which involve stabilization in either the keto-amine form (N-H…O hydrogen bond) [17] or the phenol-imine (N…H-O hydrogen bond) form [18]. In this X-ray study, it is revealed that HL, in its solid-state form, functions as a Schiff base and exists in the phenol-imine form. The bond length between C8 and N1 (1.282(3) Å) validates the presence of a double bond and is consistent with similar values found in other Schiff bases [19]. Additionally, the bond length C7-N1 (1.415(3) A°) is typical for a single bond. In the title compound, C_25_H_19_NO_2_, the pyrene ring system is inclined to the planar benzene ring system by 32.38°, and the conformation around the N=C bond is found to be E. Within the crystal structure, molecules are connected through C-H···π interactions (Figure 3).

#### 2.1.2. NMR Spectral Studies (^1^H and ^13^C NMR Spectra)

The ligand ^1^H NMR spectrum obtained in DMSO-d_6_ displays the following signals outlined in Table 4 and depicted in Figure S1 (an expanded region between 6 and 9 ppm is shown in Figure S2). The pyrene multiplets are seen at δ 8.49–8.12 ppm, and the H1 proton at δ 8.12 is seen as a triplet with a coupling constant of 8.04 Hz. The signal at δ 9.28 corresponds to the azomethine proton (-CH=N-) and appears as a singlet. The broad peak observed at δ 13.61 ppm is associated with the phenolic OH group within the ligand. Furthermore, the signal at δ 1.43 ppm, displaying a triplet pattern with *J* = 7.07 Hz, is attributed to the methyl group of the ethoxy substituent attached to the benzene ring, while the signal at 4.14 ppm, appearing as a quartet with *J* = 7.07 Hz, is assigned to the -CH_2_ protons of the ethoxy group. The ligand’s ^13^C NMR spectrum is obtained in DMSO-d_6_, and the data from this spectrum validate the findings observed in the ^1^H NMR spectrum. The number of signals in the ^13^C NMR spectrum aligns with the number of magnetically distinct carbon atoms in the ligand (Figure S3). The carbon atom of the azomethine group is detected at a chemical shift of δ 164.97 ppm. The Ar-C-N carbon, phenolic carbon, and Ar-C-OEt carbon are visible at δ 151.25, 147.61, and 142.06 ppm, respectively. Peaks in the region δ 131.46–116.80 ppm are due to the remaining pyrene and aromatic carbons. The carbon atom in the methyl group of the ethoxy substituent is detected at a chemical shift of 15.25 ppm, while the carbon atom in the methylene group is observed at δ 64.62 ppm. Thus, ^1^H and ^13^C NMR spectra confirm the formation of the ligand as proposed.

**Figure 4:**
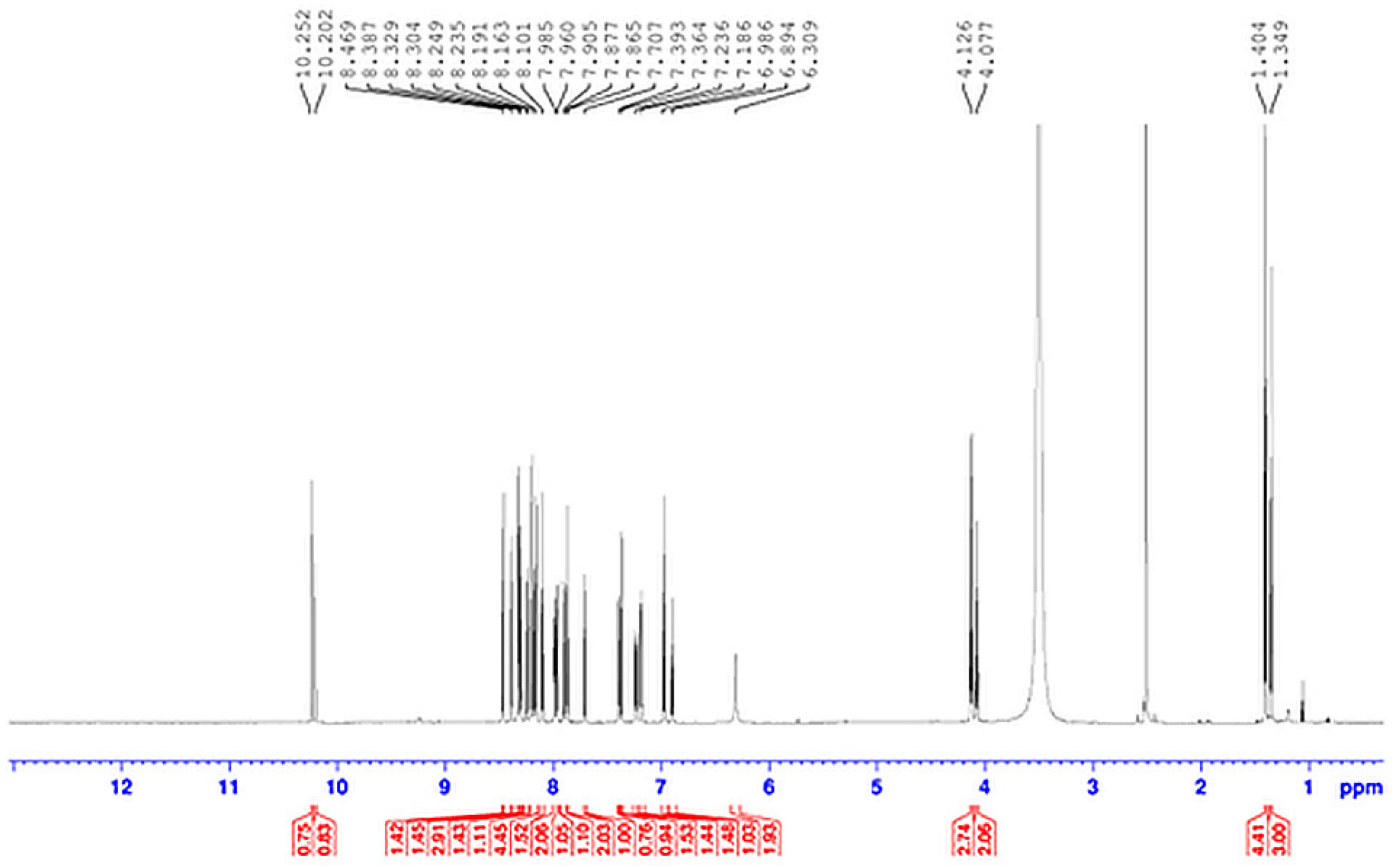
^1^H NMR spectrum of [Zn(II)(L)(Cl)(H_2_O)].

The ^1^H NMR spectrum of the [Zn(II)(L)(Cl)(H_2_O)] complex in DMSO-d_6_ shows similar signals to that observed for HL (Figure 4 and Figure S4). Regarding the Zn(II) complex, the lack of a signal stemming from the proton in the phenolic OH group provides confirmation that the phenolic oxygen atom is engaged in bonding with the metal ion through deprotonation. The signals at δ 10.25–10.20, 8.47–7.71, 7.39–6.89, 4.13–4.08, and 1.40–1.35 are due to-CH=N-protons, pyrene protons, aromatic protons, -OCH_2_ protons, and methyl protons [20]. The broad signal at δ 6.31 may be attributed to coordinated water molecules. The large difference in chemical shift values for the hydrogen atom attached to the azomethine nitrogen compared with the ligand and Zn(II) complex suggests coordination through the azomethine nitrogen in the latter. With the presence of two peaks for each signal, we believe there may be an equilibrium between different conformations in solution and protons in the two halves of the molecules that are magnetically not equivalent.

#### 2.1.3. Infrared Spectra and Coordination Mode

A comparison was made between the FTIR spectrum of the free ligand and the spectra of the complexes (Figures S5–S9). Table 5 shows the key IR bands along with their corresponding assignments. The presence of a strongly hydrogen-bonded OH group is suggested by a broad band observed at 3431 cm^−1^ in the Schiff base spectrum. This also implies that, in the solid state, the ligand is predominantly present in the enol form [21]. All metal complexes displayed broad bands within the range of 3433 to 3415 cm^−1^, signifying the existence of coordinated water molecules [20], but for Co(II)(L)_2_, this may be due to water of crystallization. The absorption band observed at 1605 cm^−1^ in the ligand’s spectrum is a result of the stretching vibration of ν(CH=N). However, in the case of the complexes, the frequency of the azomethine (CH=N) experiences a shift, either to a higher or lower field, indicating coordination through the azomethine nitrogen atom [22].

The aromatic ring ν(C=C) stretching vibration is observed in the region 1365–1492 cm^−1^. The stretching frequency ν(C-O) (phenolic) in the ligand is detected at 1252 cm^−1^ but experiences a shift to a lower frequency range within the complexes, ranging from 1208 to 1238 cm^−1^. This shift indicates bonding through the phenolic oxygen. The attachment of M(II) and M(III) atoms to the ligand via nitrogen and oxygen atoms is confirmed by the emergence of new bands at 527–626 cm^−1^ (assigned to *ν*(M − O) stretching) and 843–846 cm^−1^ (assigned to *v*(M − N) stretching) [23]. Therefore, based on the data gathered from the IR spectrum, it can be concluded that the coordination sites of the metal ion are -C=N and -CO.

#### 2.1.4. Magnetic Moments and Electronic Spectral Data

**Figure 5:**
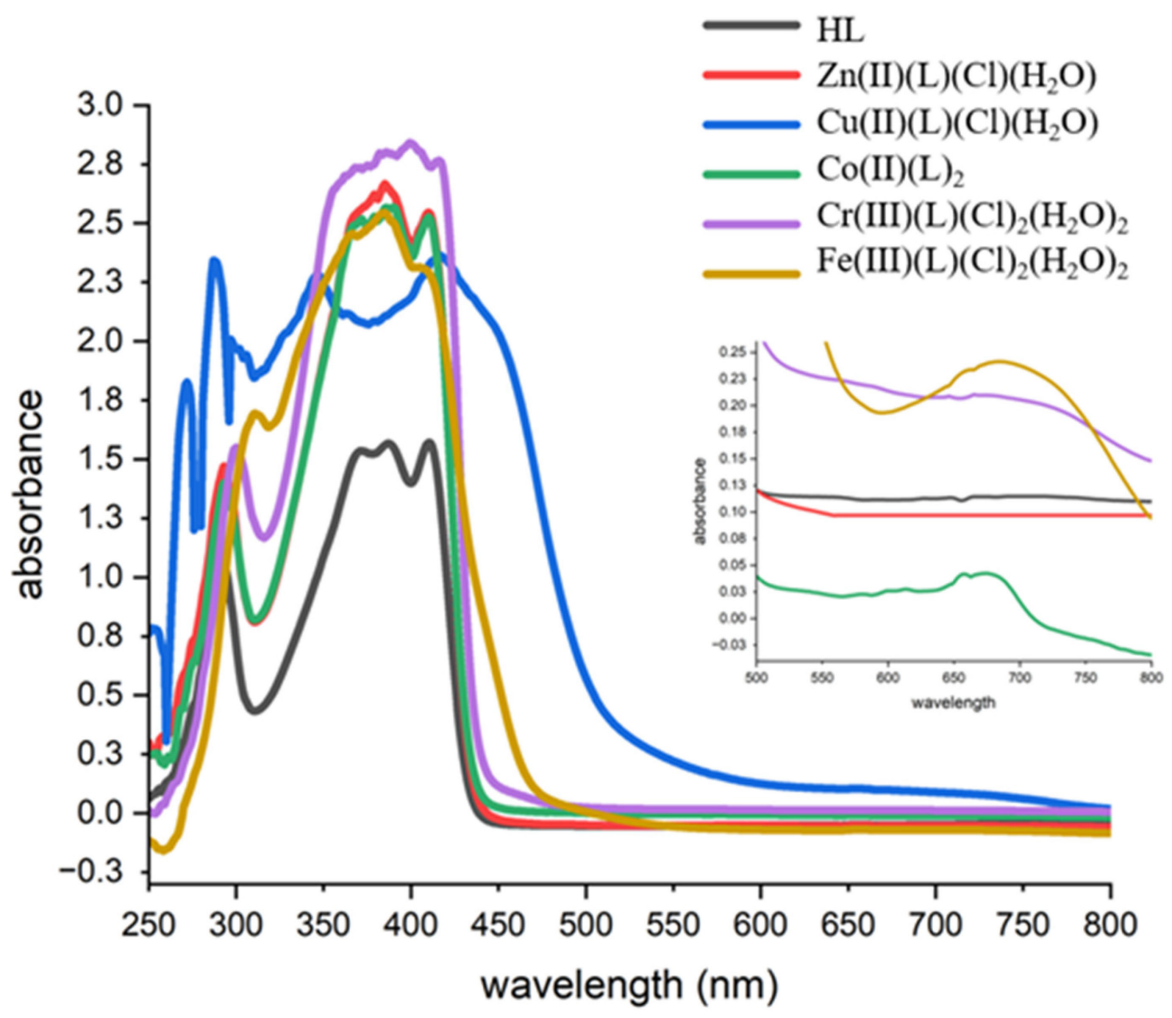
The UV-vis spectrum of HL and its metal complexes in the range of 250–800 nm.

Details about the geometry of the metal complexes were derived from their electronic absorption spectra and magnetic moment measurements, as provided in Table 6. Three bands can be observed in the electronic spectrum of the unbound Schiff base, the first two at 292 nm (34,247 cm^−1^) and 370 nm (27,027 cm^−1^) are due to the π-π* transition originating in the phenyl and pyrene rings, while the third band at 425 nm (23,529 cm^−1^) suggests the presence of n-π* transitions associated with the azomethine linkage (Figure 5) [24]. The absorption bands caused by π-π and n-π transitions in the spectrum of the free ligand, which were observed at higher energy, shifted in all spectra of metal complexes due to the coordination of the ligand with metal ions. As anticipated for a d^10^ system, the absence of d-d bands and diamagnetic behavior were observed in the Zn(II) complex. Based on conductance, analytical, and spectral data, the Zn(II) complex was assigned a tetrahedral geometry. The UV-Vis spectral data of the Cu(II) complex show two absorption bands above 347 nm, centered approximately within the ranges of 425 nm and 700 nm. It is possible that the band observed at a wavelength of 425 nm could be attributed to charge transfer from the nitrogen atoms to the copper ions [25]. The complex displays a broad band in the range of 650–750 nm, which is attributed to d-d electronic transitions (^2^T_2_ → ^2^E) and indicates the presence of Cu(II) ions in a distorted tetrahedral geometry. The magnetic moment value of the Cu(II) complex is 1.70 B.M., which is consistent with the 3d^9^ configuration having one unpaired electron in the 3d shell. A peak with a wavelength of 675 nm is observed in the electronic spectrum of the Co(II) complex. This peak is a result of the ^4^A_2_(F) → ^4^T_1_(P) transition, indicating the presence of a tetrahedral environment of the ligand surrounding the metal ion. Therefore, the geometry of the Co(II) complex can be identified as tetrahedral [26,27]. The magnetic moment value of 4.16 BM for the Co(II) complex is consistent with high-spin Co^2+^ ions, which is in agreement with the reported value for tetrahedral geometry. The electronic spectrum for the Cr(III) complex band exhibited at 680 nm is assigned to the ^4^A_2g_ → ^4^T_2g_ (F) transition, as expected for octahedral complexes [28]. The magnetic moment value of 3.61 B.M. for the Cr(III) complex indicates the presence of three unpaired electrons in its outer valence shell, creating a distorted octahedral geometry around the Cr(III) ion. Typically, electronic transitions in Fe(III) systems are considered spin forbidden and, therefore, relatively weak. However, in certain spin equilibrium systems, the high-spin (S = 5/2) configuration is associated with a transition occurring between 555 and 500 nm, and the low-spin (S = 1/2) configuration features a transition in the range of 714–625 nm. Analyzing the spectrum of the Fe(III) complex, it is evident that it displays a single band at 695 nm, which can be attributed to the ^6^A_1g_ → ^4^T_1g_ transition, a characteristic of an octahedral structure [29]. The room temperature magnetic moment of the Fe(III) complex is 5.91 B.M., which indicates that it is paramagnetic. This value was observed to be specific for octahedral complexes [30].

#### 2.1.5. Mass Spectra

**Figure 6:**
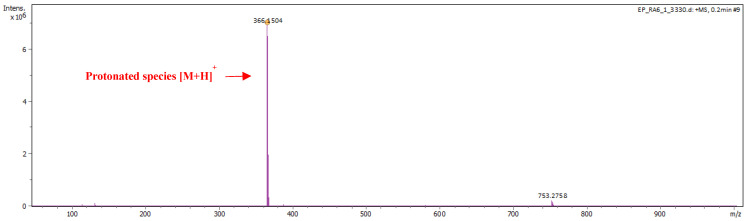
Mass spectrum of HL.

The ligand and its metal complexes were compared in terms of their stoichiometric composition using their ESI mass spectra. The data are presented in Table 7, and the spectra are displayed in Figure 6 and Figures S10–S14. A molecular ion peak was observed at *m*/*z* 366.155 for the ligand [HL]. This peak corresponds to [HL+H]^+^ (C_25_H_20_NO_2_), with a calculated *m*/*z* of 365.141. A molecular ion peak at *m*/*z* = 482.407 is observed in the mass spectrum of the Zn(II) complex, which is indicative of the [M+1]^+^ peak. Mass analysis with elemental analysis supports the structure of the complex and confirms the stoichiometry of metal chelates as of [Zn(II)(L)(Cl)(H_2_O)] type. The peak at *m*/*z* = 488.132 corresponds to an adduct of acetonitrile [M-Cl+CH_3_CN]^+^. In the spectrum of the Cu(II) complex, there is a peak observed at *m*/*z* = 519.208. This peak corresponds to an adduct of DMF, specifically [M-Cl+DMF]^+^. Mass and elemental analyses validate the complex’s structure and confirm that the metal chelates have a stoichiometry of [Cu(L)(Cl)(H_2_O)]. A peak at *m*/*z* = 788.218 is observed in the mass spectrum of the Co(II) complex, which is indicative of the [M+1]^+^ peak. Mass analysis with elemental analysis supports the structure of the complex and confirms the stoichiometry of metal chelates as of Co(L)_2_ type. The mass spectrum of the Cr(III) complex shows a peak at *m*/*z* = 432.160, which corresponds to the [M+1-2Cl-H_2_O]^+^ peak. The peak at *m*/*z* = 597.147 corresponds to an adduct of dimethyl formamide [M+2DMF-H_2_O-Cl]^+^. The structural integrity of the complex is verified, and the stoichiometry of the metal chelates is affirmed as being of the type of [Cr(L)(Cl)_2_(H_2_O)_2_] through mass and elemental analysis. The mass spectrum of the Fe(III) complex displayed a molecular ion peak at *m*/*z* = 526.84 for [M]^+^, and the peak at *m*/*z* = 599.891 corresponds to an adduct of dimethyl formamide [M+2DMF-2Cl]^+^. Mass analysis with elemental analysis supports the structure of the complex and confirms the stoichiometry of metal chelates of [Fe(III)(L)(Cl)_2_(H_2_O)_2_] type. In the mass spectra of metal complexes, various molecular ion peaks arise due to the fragmentation of the metal complex molecule. These peaks occur because of the rupture of different bonds within the molecule, leading to the formation of various radicals and many more important peaks. The molecular ion peaks observed in the spectra of these complexes correspond well with the structure proposed by spectral, magnetic, and elemental analysis studies.

#### 2.1.6. ESR Spectrum of the Cu(II) Complex

**Figure 7:**
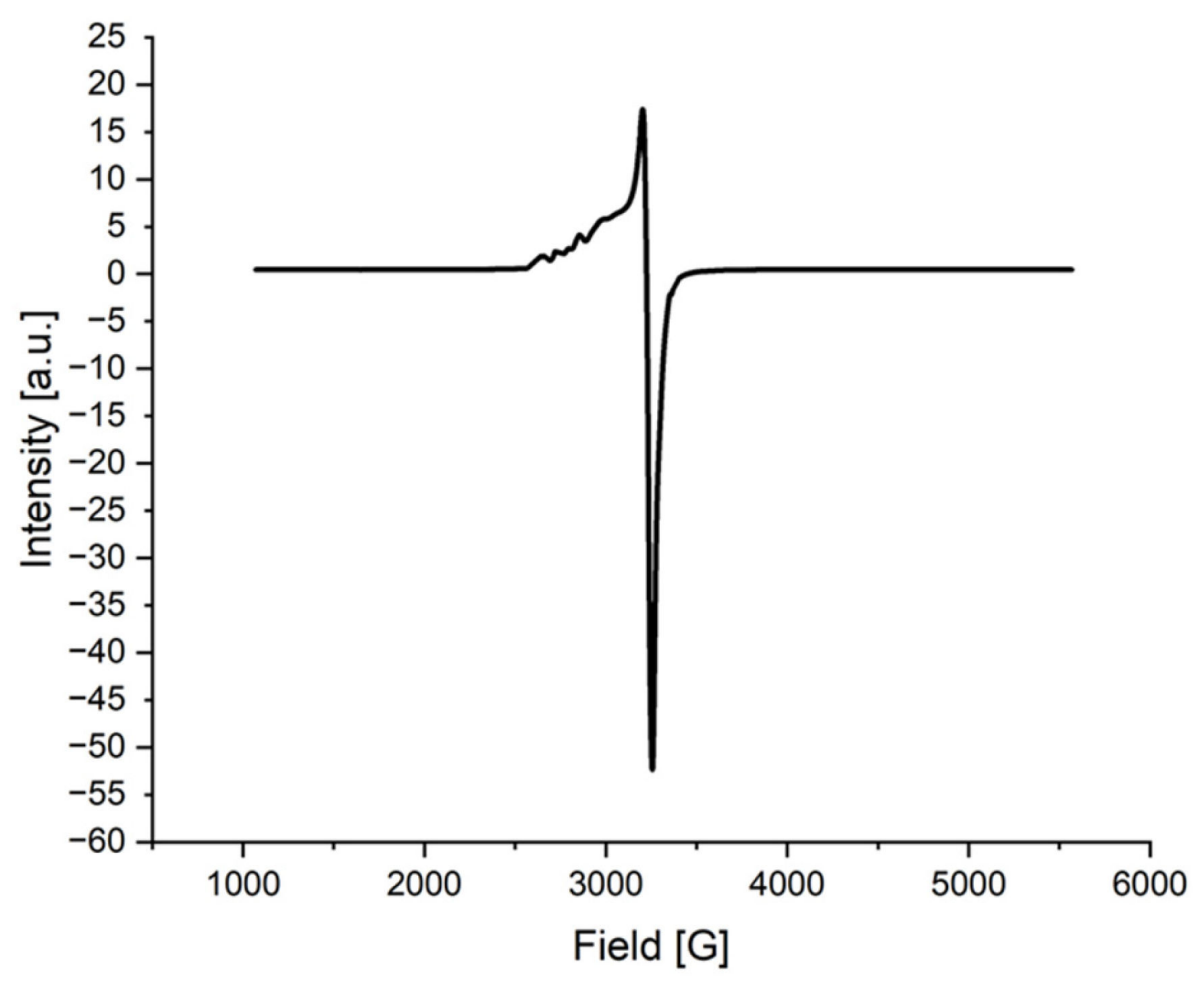
ESR spectrum of Cu(II) Schiff base complexes at 300 K.

The solid-state ESR spectrum of the [Cu(II)(L)(Cl)(H_2_O)] complex was measured at 300 K to obtain information about the geometry around the Cu metal ion. Diphenylpicrylhydrazyl (DPPH) as a g marker was used to obtain the g values. The spectrum exhibits four distinct but relatively weak peaks in the lower field region and one highly intense peak in the higher field region (see Figure 7). The magnetic susceptibility of 1.7 BM for the Cu(II) complex indicates the presence of a single unpaired electron, confirming the complex’s mononuclear nature. This conclusion is further supported by the absence of a half-field signal at 1600 G in the ESR spectrum, which rules out any Cu–Cu interactions [26]. The observed g values are as follows: g∥ (2.49) > g_⊥_ (2.18) > ge (2.0023), implying that the unpaired electron resides in the dx^2^-y^2^ orbital. The g∥/A∥ values of square planar complexes were reported within the range of 105–135 cm, whereas tetrahedral distorted complexes were in the range of 150–250 cm. The g∥/A∥ value of the Cu(II) complex of 205.79 cm is in the range expected for distorted tetrahedral rather than square planar complexes [14]. In the axial spectra, the g values are associated with the exchange interaction coupling constant (G). The observed G value of 2.72 in the current copper complex (where G < 4.0) indicates the presence of substantial exchange coupling, and there is a noticeable misalignment [26]. The covalency parameter α^2^, related to in-plane σ bonding as per Equation (1), was determined to be 0.51, suggesting the presence of covalent bonding. (An α^2^ value of 0.5 implies complete covalent bonding, while a value of 1.0 suggests complete ionic bonding.)

α^2^ = −(A∥ = 0:036) + (g∥ − 2.0036) + 3/7(g_⊥_ − 2.0036) + 0.04 (1)

### 2.2. Cytotoxic Activity

The cytotoxic effects of the newly synthesized Schiff base and its complexes were evaluated in vitro using the MTT colorimetric assay on the MCF-7 human breast cancer cell line (Table 8 and Table 9). The results include IC_50_ values, which represent the concentration (in μg/mL) of a compound required to induce 50% cell death compared with the control culture. As a reference, fluorouracil (5FU) was used. Notably, the MCF-7 cells displayed sensitivity to the Schiff base HL, with an IC_50_ value of 63.901 ± 3.67 μg/mL. The Cu(II) complex is the most cytotoxic, with an IC_50_ value of 5.661 ± 0.33 μg/mL, followed by the Zn(II) complex. The Cu(II) complex is the most effective compound, even more than the 5FU drug, followed by the Zn(II) complex and then the Fe(III) complex. All the complexes exhibit greater effectiveness against the human breast cancer cell line compared with the Schiff base ligand. The bonding of metal ions enhances the anticancer properties, as indicated by the complexes having lower IC_50_ values in comparison with the uncoordinated ligand.

## 3. Experimental Section

### 3.1. Materials and Instrumentation

We used commercially purchased reagents and solvents without further purification. 3-ethoxy salicylaldehyde and 1-aminopyrene were purchased from Sigma–Aldrich (Burlington, MA, USA). Zinc chloride hexahydrate (ZnCl_2_·6H_2_O), copper chloride dihydrate (CuCl_2_·6H_2_O), cobalt chloride hexahydrate (CoCl_2_·6H_2_O), chromium chloride hexahydrate (CrCl_3_·6H_2_O), and iron chloride anhydrous (FeCl_3_) were purchased from BDH chemicals (London, UK).

Elemental analyses were performed using a Thermo Flash 2000 CHN Elemental analyzer (Thermo Fisher Scientific, Waltham, MA, USA). Infrared spectra for the Schiff base ligand and its metal complexes were recorded in KBr pellets, employing a Perkin Elmer FT-IR Spectrometer within the range of 4000–400 cm^−1^ (Perkin Elmer, Inc., Waltham, MA, USA). Electronic spectra were recorded in DMSO for the ligand and its complexes in the range of 200–800 nm using a MultiSpec-1501 UV-VIS spectrophotometer (Shimadzu, Kyoto, Japan). These spectra were obtained at room temperature using 1.0 cm of quartz. ^1^H and ^13^C NMR spectra were scanned using an 850 MHz Brucker Ascend Spectrophotometer. Solid-state paramagnetic resonance (EPR) analysis was conducted using the continuous wave Bruker EMX PLUS spectrometer (Bruker BioSpin, Rheinstetten, Germany). The molar conductance measurements were carried out at room temperature using an OHAUS-STARER 3100C conductivity meter with a 0.001 M solution of complexes in DMSO (OHAUS Corporation, Parsippany, NJ, USA). The room temperature magnetic susceptibility was measured using Sherwood Scientific Magnetic susceptibility balance (Sherwood Scientific Ltd., Cambridge, UK).

### 3.2. Crystal Structure Determination of HL

The single-crystal X-ray data were gathered at a temperature of 150 K using a Bruker APEX-II CCD diffractometer. A graphite-monochromated MoKα radiation source (wavelength λ = 0.71073 A) and the w-scan technique were employed. The structures were initially solved through direct methods utilizing SHELXT [31] and subsequently refined using the full matrix least-squares method with SHELX integrated into the OLEX2 program suite v.1.2. Anisotropic displacement parameters were applied in the refinement of non-hydrogen atoms. All hydrogen atoms were determined from a different Fourier map and refined isotropically. Data collection: Bruker APEX2; cell refinement: Bruker SAINTl; and data reduction: Bruker SAINT. The general-purpose crystallographic tool PLATON v.2023.1 was used for the structure analysis [32]. MERCURY v.3.8 [33] and ORTEP-3 v.1.0.3 [34] were employed for the structural analysis and for presenting the results.

### 3.3. Synthesis of the Ligand (E)-2-Ethoxy-6-((pyren-1-ylimino)methyl)phenol (HL)

The 3-Ethoxysalicylaldehyde (0.083 g, 5 mmol) was dissolved in 30 mL of ethanol and, after complete dissolution, an equimolar amount of 1-aminopyrene (0.109 g, 5 mmol) dissolved in 30 mL of ethanol was added. Three drops of acetic acid anhydride were introduced into the reaction mixture as a catalyst. Subsequently, the solution was subjected to reflux for a duration of three hours, after which it was allowed to cool down to room temperature. The resulting precipitate was then collected and washed with ethanol to obtain the Schiff base, referred to as HL. Crystals of HL were obtained via slow evaporation of the dissolved compound in ethanol. Color: orange; yield: 96.69% (1.77 g); m.p.: 105 °C. Anal. calc., (C_25_H_19_NO_2_) C, 82.2; H, 5.2; N, 3.8 found, C,82.3; H, 5.3; N, 3.9. ^1^H NMR (850 MHz, DMSO, ppm) δ 13.61 (s, 1H, -OH), 9.28 (s, 1H, -CH=N-), 8.49 (d, 1H, *J* = 6.4 Hz, pyrene), 8.41 (d, 1H, *J* = 8.84 Hz, pyrene), 8.33 (m, 2H, pyrene), 8.32 (d,1H, *J* = 9.49 Hz, pyrene), 8.22–8.18 (m, 3H, pyrene), 8.12 (t, 1H, *J* = 8.33 Hz, pyrene), 7.42 (d, *J* = 9.1 Hz, 1H, Ar-H), 7.21 (d, *J* = 7.2 Hz, 1H, Ar-H), 6.99 (t, *J* = 8.04 Hz, 1H, Ar-H), 4.14 (q, *J* = 7.07 Hz, 2H, -OCH_2_), 1.43 (t, *J* = 7.07, 3H, -CH_3_). ^13^C NMR (850 MHz, DMSO, ppm) δ 164.97 (-CH=N-), 151.25 (Ar-C-N), 147.61 (Ar-C-OH), 142.06 (Ar-C-OEt), 131.46–116.80 (Ar-C), 64.26 (CH_2_), 15.25 (CH_3_).

### 3.4. Synthesis of the Schiff Base Metal Complexes

The prepared metal complexes were synthesized as follows: the Schiff base HL (0.731 g, 2 mmol) was dissolved in 40 mL of ethanol and combined with an ethanolic solution of ZnCl_2_·6H_2_O, CuCl_2_·6H_2_O, and CoCl_2_·6H_2_O (2 mmol, 20 mL). For the Cr(III) and Fe(III) complexes, a solution of HL was dissolved in 20 mL of ethanol and then added to an ethanolic solution of CrCl_3_·6H_2_O and FeCl_3_ (1 mmol, 10 mL). The resulting solutions were stirred magnetically and refluxed for three hours. After cooling, the resulting precipitates were collected using filtration, washed several times with hot ethanol, and dried. All the complexes were stable in the presence of air and moisture. The data regarding the analysis and physical properties of the ligand (HL) and the metal complexes can be found in Table 1.

For [Zn(II)(L)(Cl)(H_2_O)], a red solid was obtained; m.p.: 215 °C; yield (22.9%, 0.22 g). ^1^H NMR (850 MHz, DMSO, ppm) δ 10.25, 10.20 (s, 1H, -CH=N-), 8.50–7.71 (m, 9H, pyrene), 7.39–6.89 (3H Ar-H), 6.31 (s, 2H, Zn-H_2_O), 4.14, 4.09 (q, 2H, -OCH_2_), 1.4, 1.35 (t, 3H, -CH_3_).

For [Cu(II)(L)(Cl)(H_2_O)], a black solid was obtained; m.p.: >250 °C with decomposition; yield (72.5%, 0.696 g).
For [Co(II)(L)_2_], a dark red solid was obtained; m.p.: >250 °C with decomposition; yield (44.0%, 0.694 g).
For [Cr(III)(L)(Cl)_2_(H_2_O)_2_], a pale brown solid was obtained; m.p.: >250 °C with decomposition; yield (16.9%, 0.090 g).
For [Fe(III)(L)(Cl)_2_(H_2_O)], a black solid was obtained; m.p.: >250 °C with decomposition; yield (49.59%, 0.261 g).

### 3.5. Sample Preparation and Analysis for Mass Analysis

The samples were dissolved in DMF. An approximately 10 microliter aliquot of the dissolved sample was further diluted 1000-fold in acetonitrile. No precipitation or cloudiness was observed. Analysis was performed via flow injection whereby 5 mL of each sample was injected into the LC flow of 100 acetonitrile mobile phase composition and directly introduced into the MS electrospray ionization source for ionization generation. The experiments were conducted on a QTOF MS using an ESI ionization source. The MS data were externally calibrated using calibration data of sodium formate clusters.

### 3.6. Cell Viability Test

Cell viability was evaluated through the MTT assay using mononuclear cells. This method is based on the reduction of a soluble yellow tetrazolium salt into insoluble purple formazan crystals by metabolically active cells. Within the mitochondria of live cells, the enzyme mitochondrial succinate dehydrogenase transforms the internalized tetrazolium salt into purple formazan crystals. The cells are then lysed and dissolved in a DMSO solution. The resulting color change is measured using an ROBONIK P2000 Elisa Reader at 570 nm (India PVT LTD., Thane, India). For the assay, the MCF-7 human breast cancer cell line is plated separately in 96-well plates at a concentration of 1 × 10^5^ cells per well. After 24 h, the cells are washed twice with 100 μL of serum-free medium and starved for an hour at 37 °C. Subsequently, the cells are treated with varying concentrations of the test compound (ranging from 0.4 to 100 μg/mL) for 24 h. At the end of the treatment period, the medium is removed, and a serum-free medium containing MTT (0.5 mg/mL) is added, followed by incubation for 4 h at 37 °C in a CO_2_ incubator. The MTT-containing medium is then discarded, the cells are washed with PBS (200 μL), and the crystals are dissolved by adding 100 μL of DMSO. This solution is thoroughly mixed via pipetting. The spectrophotometric absorbance of the purple–blue formazan dye is measured using a microplate reader at 570 nm [35,36].

## 4. Conclusions

In this work, we synthesized a novel pyrene-based Schiff base by reacting 3-ethoxy salicylaldehyde and 1-aminopyrene and conducted a detailed analysis of its crystallographic data. Furthermore, we synthesized and characterized Zn(II), Cu(II), Co(II), Cr(III), and Fe(III) complexes of this Schiff base using various techniques, including elemental analysis, mass spectrometry, infrared spectroscopy, and magnetic measurements. The results clearly demonstrate the bidentate nature of the Schiff base ligand. In all of these complexes, the metal-to-ligand stoichiometry is 1:1, with the exception of the Co(II), which exhibits a 1:2 ratio. Electronic and ESR spectral studies suggest distorted tetrahedral geometry for the Cu(II) complex. The Zn(II) and Co(II) complexes adopt tetrahedral geometry, while the Cr(III) and Fe(III) complexes exhibit distorted octahedral geometry. We also assessed the cytotoxicity of these metal complexes and found that the [Cu(II)(L)(Cl)(H_2_O)] complex exhibited a superior cytotoxic effect on MCF-7 cancer cells, with an IC_50_ value of 5.661 ± 0.33 μg/mL. This IC_50_ value is significantly lower than that of the standard cancer drug fluorouracil (IC_50_ = 18.047 ± 1.04 μg/m). These results indicate that the Cu(II) complex may have the potential to be utilized as a traditional anticancer agent. However, further comprehensive in vivo experiments are necessary to substantiate its anticancer effectiveness.

## Figures and Tables

**Table 1:** Analytical data and physical properties of ligand HL and its complexes.

Compounds	Empirical Formula	Mol. wt. (g/mol)	Color	M.p. (°C)	Yield (%)	Elemental Found (calc.)			Ω (O hm^−1^ cm^2^ mol^−1^)
						C	H	N	
HL	C_25_H_19_NO_2_	365.4	Orange	105	96.69	82.3 (82.2)	5.3 (5.2)	3.9 (3.8)	-
Zn(II)(L)(Cl)(H_2_O)	C_25_H_20_ClNO_3_Zn	481.0	red	215	22.93	61.9 (62.1)	4.2 (4.2)	3.1 (2.9)	2.05
Cu(II)(L)(Cl)(H_2_O)	C_25_H_20_ClNO_3_Cu	480.0	Black	>250	72.5	62.3 (62.4)	3.9 (4.2)	3.0 (2.9)	5.53
Co(II)(L)_2_	C_50_H_36_N_2_O_4_Co	787.8	Dark red	>250	44.0	75.9 (76.2)	4.5 (4.6)	3.6 (3.6)	12.4
Cr(III)(L)(Cl)_2_(H_2_O)_2_	C_25_H_22_Cl_2_NO_4_Cr	533.0	Pale Brown	>250	16.9	57.6 (57.4)	4.2 (4.3)	2.8 (2.7)	15.15
Fe(III)(L)(Cl)_2_(H_2_O)_2_	C_25_H_22_Cl_2_NO_4_Fe	526.0	Black	>250	49.59	57.2 (57.0)	4.1 (4.2)	2.6 (2.7)	10.15

**Table 2:** Crystal data and structure refinement of HL.

Crystal Data	
Chemical formula	C_25_H_19_NO_2_
*M*_r_	365.41
Crystal system, space group	Triclinic, *P*1
Temperature (K)	150
*a*, *b*, *c* (Å)	7.0543 (13), 11.503 (2), 11.988 (2)
α, β, γ (°)	104.225 (7), 97.308 (7), 102.335 (8)
*V* (Å^3^)	904.4 (3)
*Z*	2
Radiation type	Mo *K*α
µ (mm^−1^)	0.09
Crystal size (mm)	× ×
Data collection	
Diffractometer	Bruker *APEX*-II CCD
Absorption correction	–
No. of measured, independent, and observed [*I* > 2σ(*I*)] reflections	20,633, 3078, 2033
*R*_int_	0.110
(sin θ/λ)_max_ (Å^−1^)	0.588
Refinement	
*R*[*F*^2^ > 2σ(*F*^2^)], *wR*(*F*^2^), *S*	0.060, 0.132, 1.08
No. of reflections	3078
No. of parameters	256
H-atom treatment	H-atom parameters constrained
Δρ_max_, Δρ_min_ (e Å^−3^)	0.22, −0.20

**Table 3:** Selected bond lengths (Å) and bond angles (°) for HL.

Bond Lengths (Å)		Bond Lengths (Å)	
O1-C11	1.372 (3)	C11-O1-C12	117.5 (2)
O1-C12	1.443 (3)	C8-N1-C7	122.0 (2)
O2-C10	1.353 (3)	C6-C7-N1	117.3 (2)
N1-C7	1.415 (3)	C19-C7-N1	122.6 (2)
N1-C8	1.282 (3)	N1-C8-C9	121.2 (3)
O1-C11	1.372 (3)	O2-C10-C9	122.3 (2)
O1-C12	1.443 (3)	O2-C10-C11	117.7 (2)
N1-C8	1.282 (3)	O1-C12-C13	106.9 (2)
C11-C14	1.374 (4)		
C12-C13	1.503 (4)		
C14-C15	1.407 (4)		

**Table 4:** ^1^H NMR spectral data of the ligan HL.

Chemical Shift (δ, ppm)	Multiplicity	Number of Protons	Functional Group Assigned
13.61	Singlet	1	-OH
9.28	Singlet	1	-CH=N-
8.49–8.12	Multiplet	9	Pyrene protons
7.42–6.99	Doublet	3	Aromatic protons
4.14	Quartet	2	-OCH_2_
1.43	Triplet	3	-CH_3_

**Table 5:** Characteristic IR bands (cm^−1^) of HL and its metal complexes.

Compound	*v*OH/ *v*H_2_O	*v*C=N	*v*C=C	*v*C-O	*v*C-N	*v*M-N	*v*M-O
HL	3431	1605	1459	1252	1186	-	-
Zn(II)(L)(Cl)(H_2_O)	3415	1631	1492	1235	1118	843	608
Cu(II)(L)(Cl)(H_2_O)	3416	1596	1365	1208	1132	844	613
Co(II)(L)_2_	3415	1631	1492	1234	1118	843	626
Cr(III)(L)(Cl)_2_(H_2_O)_2_	3420	1738	1365	1228	1216	846	527
Fe(III)(L)(Cl)_2_(H_2_O)_2_	3433	1629	1482	1238	1189	845	607

**Table 6:** Magnetic moments and electronic spectral data for complexes.

Ligand and Complexes	V_max_ (nm)	µ_eff._ (B.M)	Geometry	d*^n^*
HL	292, 370, 425	-	-	-
Zn(II)(L)(Cl)(H_2_O)	295, 380, 430	Diamagnetic	Tetrahedral	d^10^
Cu(II)(L)(Cl)(H_2_O)	275, 347, 425, 700	1.70	Distorted tetrahedral	d^9^
Co(II)(L)_2_	293, 390, 410, 675	4.16	Tetrahedral	d^7^
Cr(III)(L)(Cl)_2_(H_2_O)_2_	300, 399, 625, 725	3.61	Distorted octahedral	d^3^
Fe(III)(L)(Cl)_2_(H_2_O)_2_	311, 385, 695	5.91	Distorted octahedral	d^5^

*n*—number of electrons.

**Table 7:** Mass spectral data of the ligand and its complexes.

Compound	Expected *m*/*z*	Found *m*/*z*	Peak Assigned
HL	365	366	[M+1]^+^
Zn(II)(L)(Cl)(H_2_O)	481	482	[M+1]^+^
Cu(II)(L)(Cl)(H_2_O)	480	519	[M-Cl+DMF]^+^
Co(II)(L)_2_	787	788	[M+1]^+^
Cr(III)(L)(Cl)_2_(H_2_O)_2_	522	432	[M+1-2Cl-H_2_O]^+^
Fe(III)(L)(Cl)_2_(H_2_O)_2_	526	527	[M+1]^+^

**Table 8:** The percentage of cell viability after treatment of MCF-7 cells with different concentrations of samples.

Sample	100 μg	25 μg	6.3 μg	1.6 μg	0.4 μg
HL	46.88	56.88	64.25	76.44	83.44
Zn(II)(L)(Cl)(H_2_O)	38.03	47.13	52.75	64.16	68.00
Cu(II)(L)(Cl)(H_2_O)	29.44	40.36	49.30	59.01	68.46
Co(II)(L)_2_	35.06	49.50	62.81	72.06	79.94
Cr(III)(L)(Cl)_2_(H_2_O)_2_	45.81	56.54	65.30	75.32	84.14
Fe(III)(L)(Cl)_2_(H_2_O)_2_	38.65	46.52	56.04	66.85	76.13
5FU ^b^	37.63	47.40	59.44	66.00	76.15

^b^ Fluorouracil cancer drug used as a positive control.

**Table 9:** In vitro antiproliferative activities of HL and its metal complexes against the MCF-7 human tumor cell line.

Compound	Cytotoxicity IC_50_^a^ μg/mL	SD ±
HL	63.901	3.67
Zn(II)(L)(Cl)(H_2_O)	12.742	0.73
Cu(II)(L)(Cl)(H_2_O)	5.661	0.33
Co(II)(L)_2_	21.141	1.21
Cr(III)(L)(Cl)_2_(H_2_O)_2_	58.708	3.37
Fe(III)(L)(Cl)_2_(H_2_O)_2_	16.895	0.97
5FU ^b^	18.047	1.04

^a^ The concentration that causes a 50% cell proliferation inhibition. ^b^ Fluorouracil cancer drug used as a positive control.

## Data Availability

Not applicable.

## Supplementary Materials

The following supporting information can be downloaded at: https://www.mdpi.com/article/10.3390/molecules28217352/s1, Figure S1: ^1^H NMR spectrum of the ligand HL; Figure S2: Expanded region between 6 and 9 ppm for the ^1^H NMR of HL; Figure S3: ^13^C NMR spectrum of the ligand HL; Figure S4: Expanded region between 6 and 9 ppm for the ^1^H NMR of [Zn(II)(L)(Cl)(H_2_O)]; Figure S5: IR spectrum of HL; Figure S6: IR spectrum of [Zn(II)(L)(Cl)(H_2_O)]; Figure S7: IR spectrum of [Cu(II)(L)(Cl)(H_2_O)]; Figure S8: IR spectrum of [Co(II)(L)_2_]; Figure S9: IR spectrum of [Fe(III)(L)(Cl)_2_(H_2_O)_2_]; Figure S10: Mass spectrum of [Zn(II)(L)(Cl)(H_2_O)]; Figure S11: Mass spectrum of [Cu(II)(L)(Cl)(H_2_O)]; Figure S12: Mass spectrum of [Co(II)(L)_2_]; Figure S13: Mass spectrum of [Cr(III)(L)(Cl)_2_(H_2_O)_2_]; Figure S14: mass spectrum of [Fe(III)(L)(Cl)_2_(H_2_O)_2_].
