# Supplementary material for: Novel Schiff Base Derived from Amino Pyrene: Synthesis, Characterization, Crystal Structure Determination, and Anticancer Applications of the Ligand and Its Metal Complexes
Source: Molecules. 2023 Oct 30;28(21):7352. doi: 10.3390/molecules28217352 (PMC10648749; doi:10.3390/molecules28217352)
Supplement: Supplementary file 1 [file molecules-28-07352-s001.zip › molecules-2671403-supplementary.pdf]

Supplementary Material

Novel Schiff Base Derived from Amino Pyrene: Synthesis, Characterization, Crystal Structure Determination, and Anticancer Applications of the Ligand and Its Metal Complexes

Elham S. Aazam * and Maryam A. Majrashi

Department of Chemistry, King Abdulaziz University, P.O. Box 23622, Jeddah, Saudi Arabia

* Correspondence: eazam@kau.edu.sa

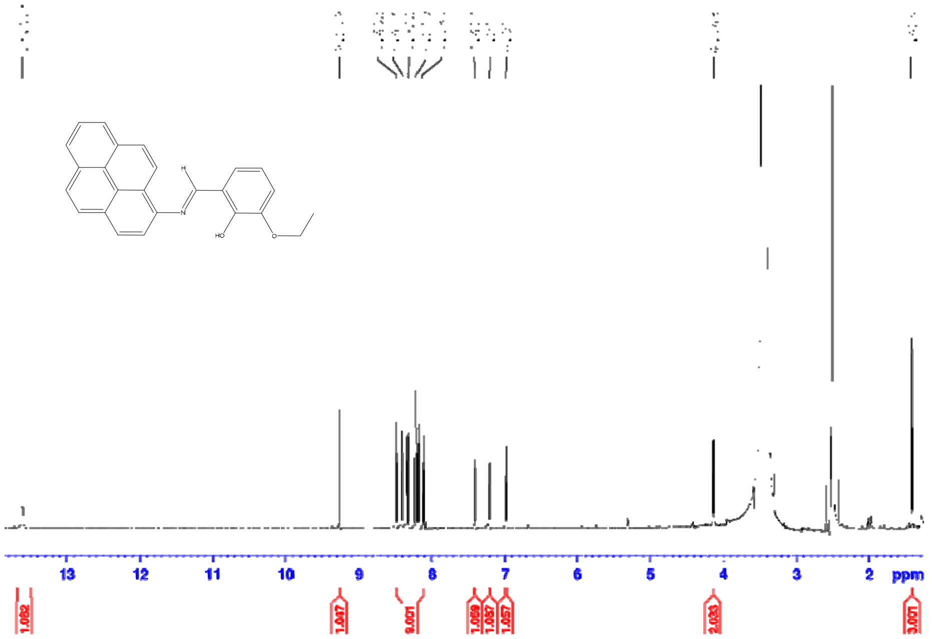

Figure S1. ^{1}H NMR Spectrum of the ligand HL.

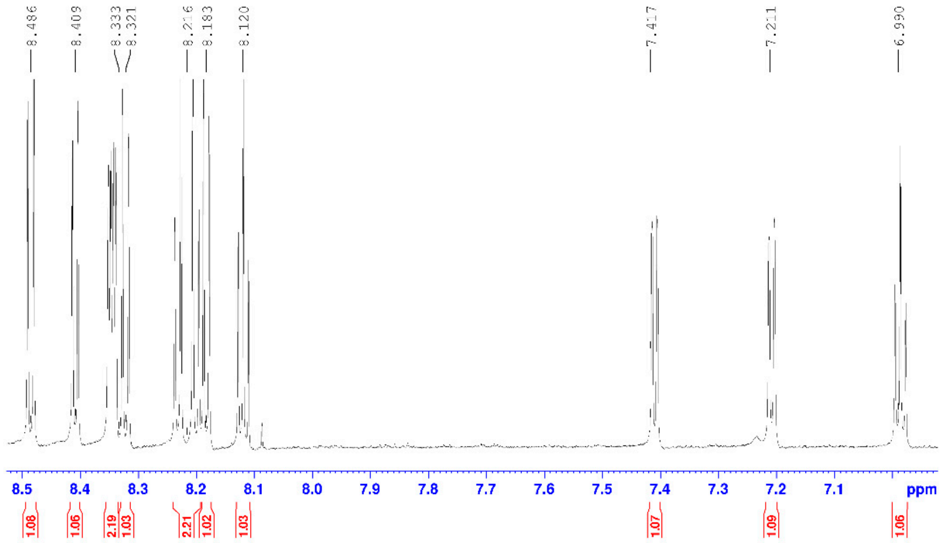

Figure S2. Expanded region between 6 and 9 ppm for the ^{1}H NMR of HL.

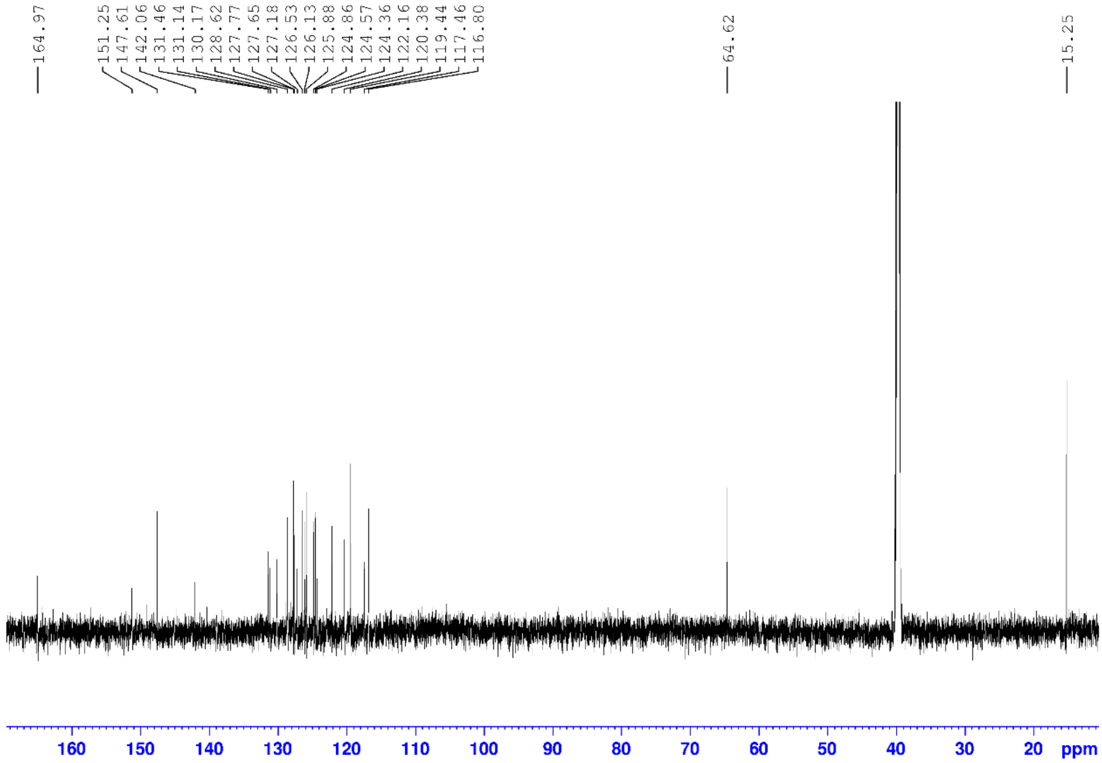

Figure S3. ^{13}C NMR Spectrum of the ligand HL.

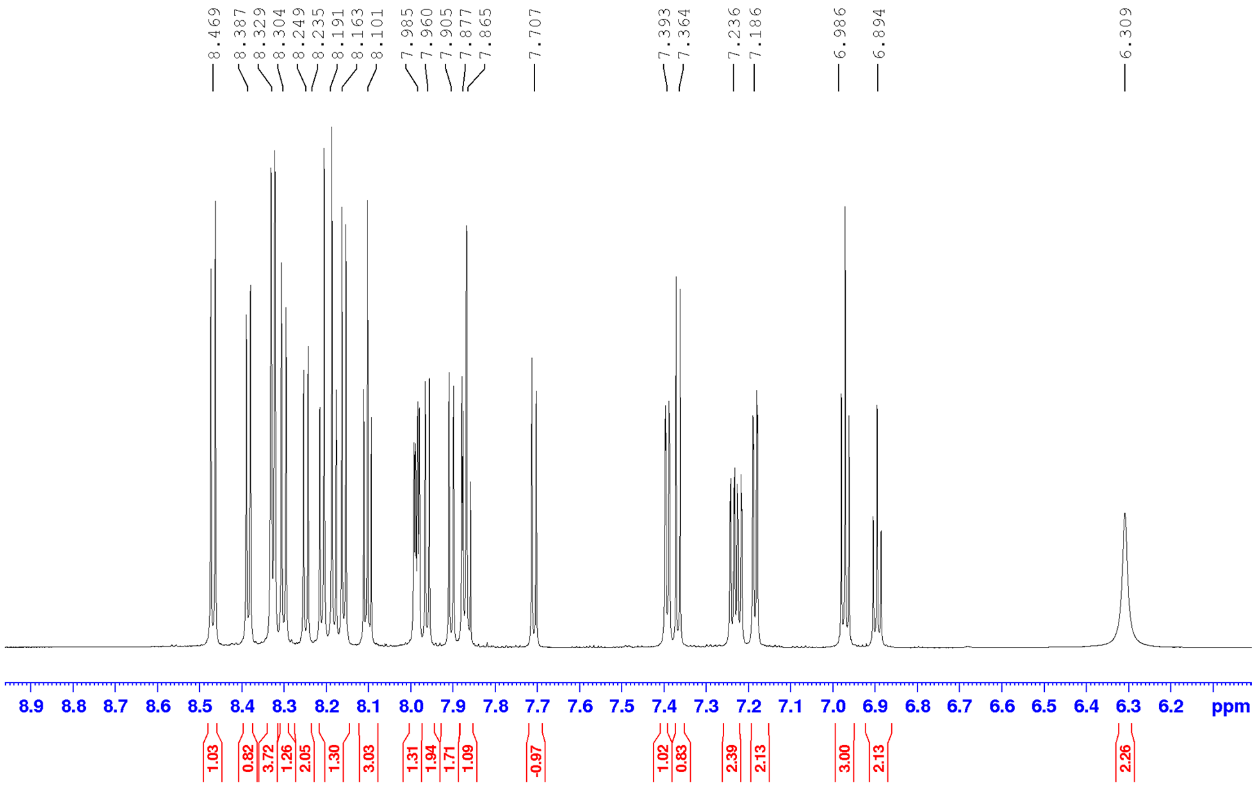

Figure S4. Expanded region between 6 and 9 ppm for the ^{1}H nmr of [Zn(II)(L)(Cl)(H_2O)].

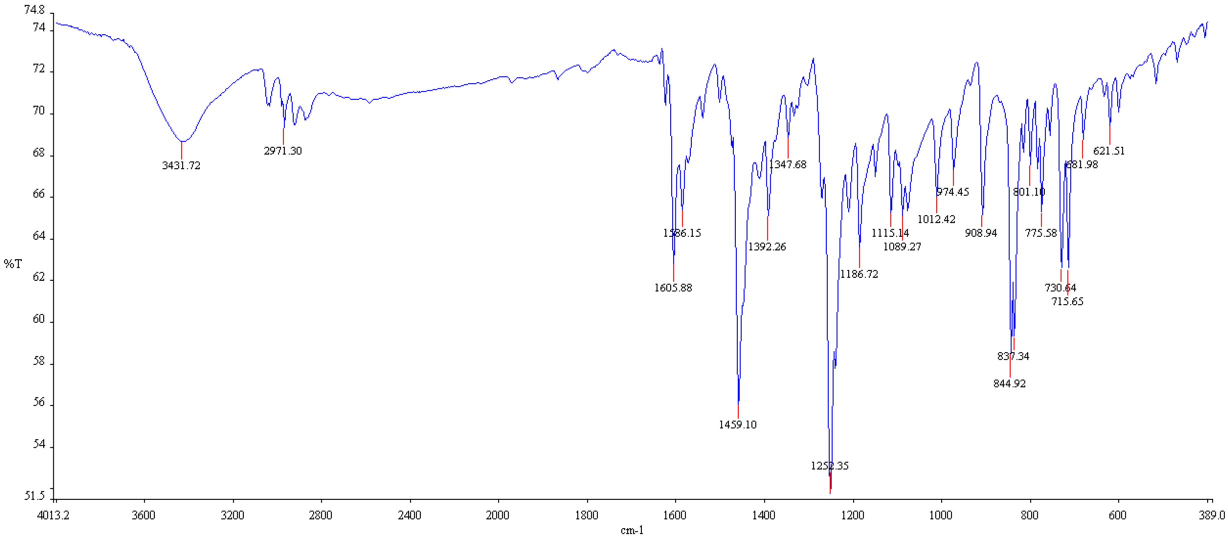

Figure S5. IR spectrum of HL.

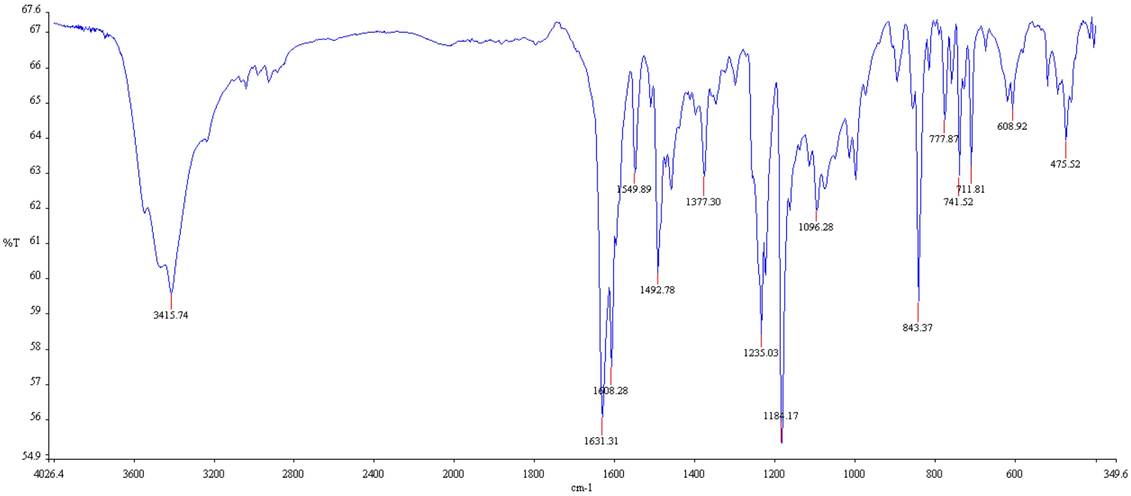

Figure S6. IR spectrum of [Zn(II)(L)(Cl)(H_2O)].

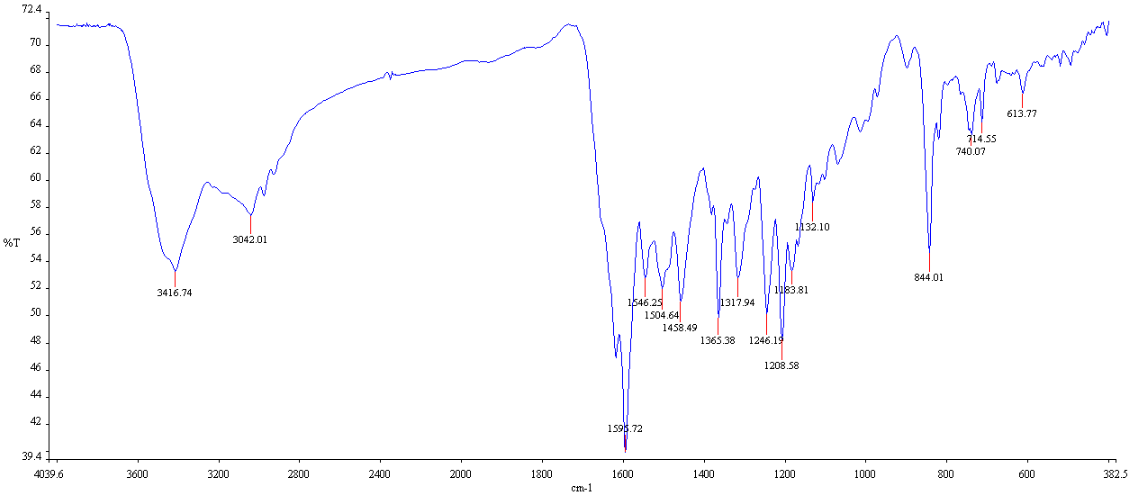

Figure S7. IR spectrum of [Cu(II)(L)(Cl)(H_2O)].

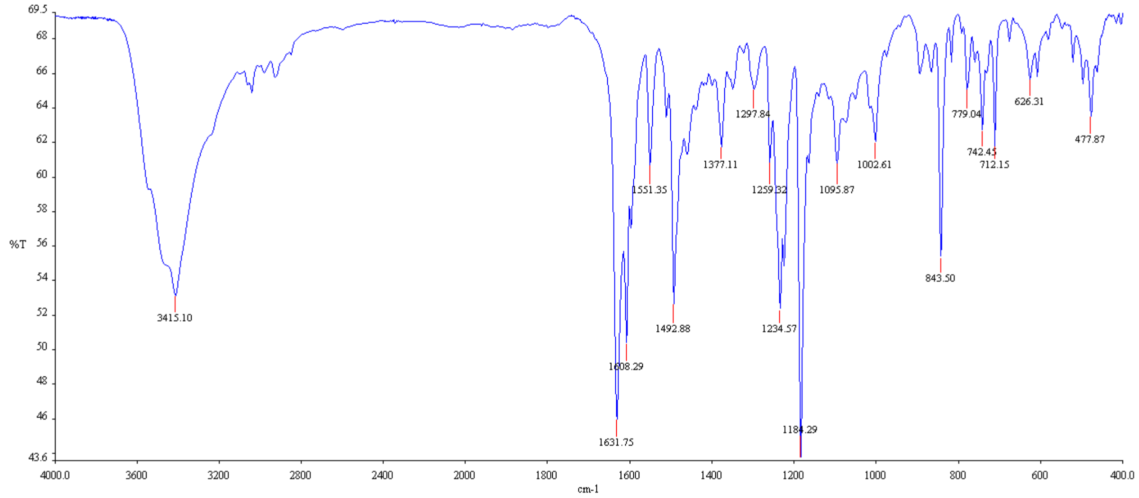

Figure S8. IR spectrum of [Co(II)(L)$_2$].

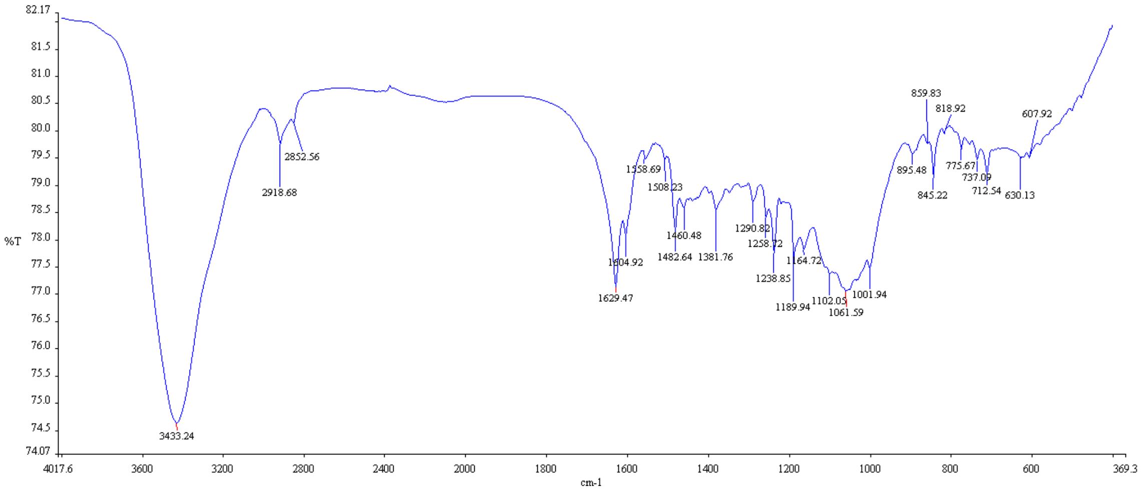

Figure S9. IR spectrum of [Fe(III)(L)(Cl)$_2$(H$_2$O)$_2$].

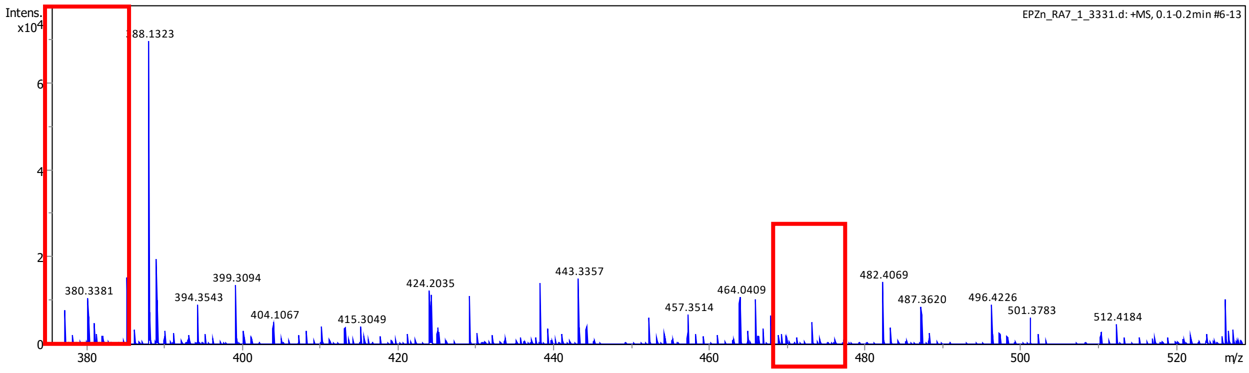

Figure S10. Mass spectrum of [Zn(II)(L)(Cl)(H$_2$O)].

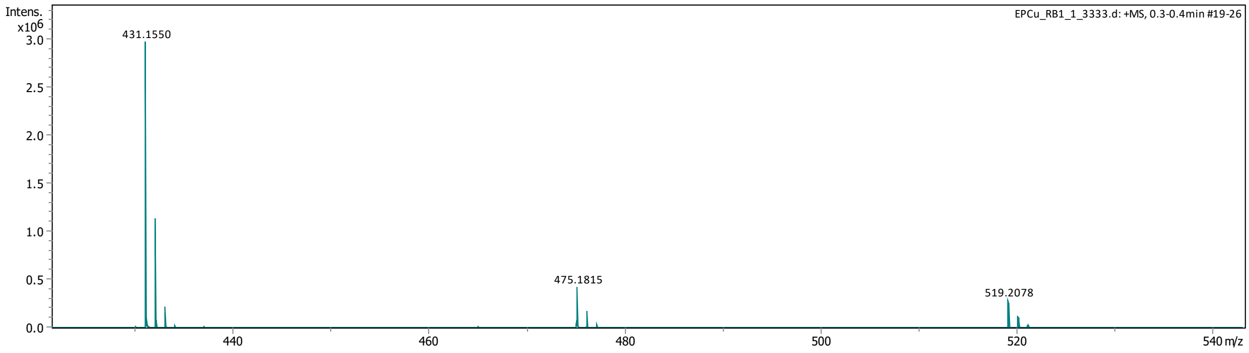

Figure S11. Mass spectrum of [Cu(II)(L)(Cl)(H_2O)].

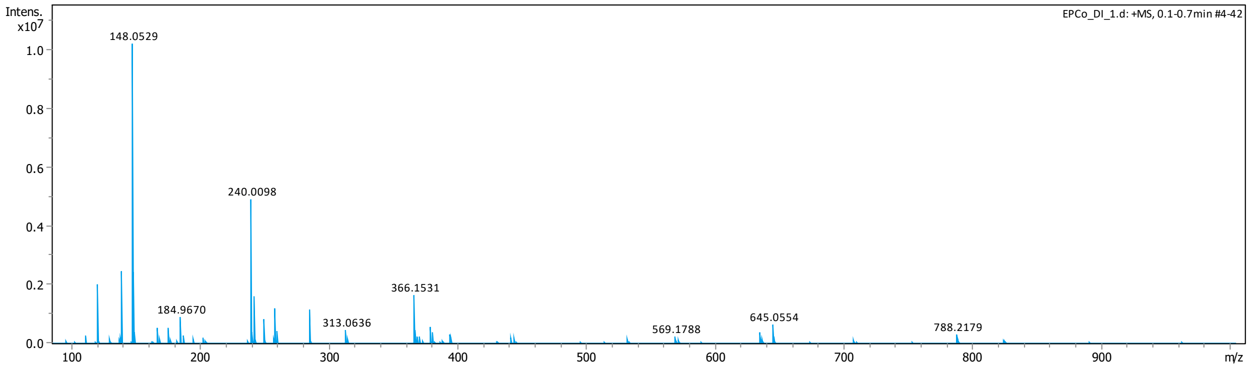

Figure S12. Mass spectrum of [Co(II)$(L)_2$].

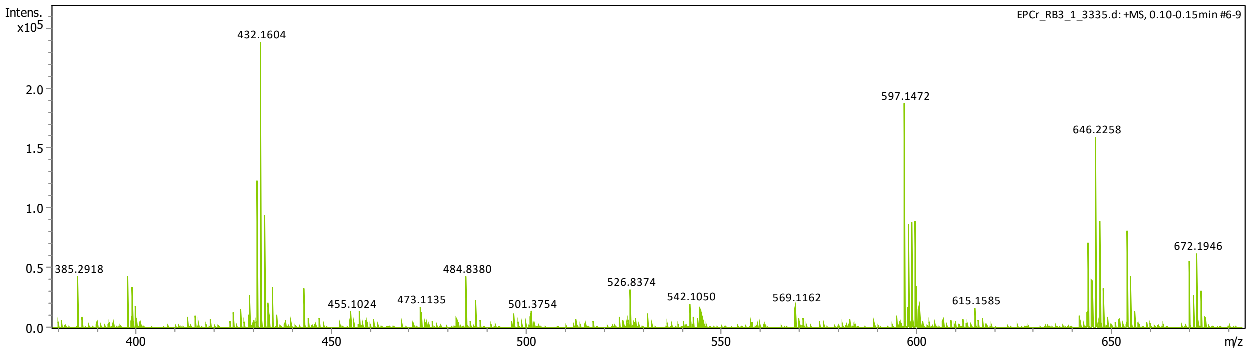

Figure S13. Mass spectrum of [Cr(III)(L)$(Cl)_2$$(H_2O)_2$].

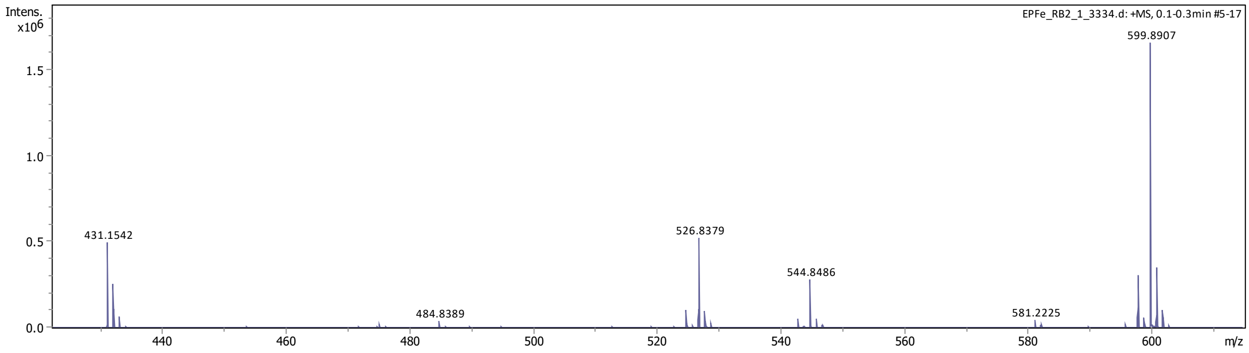

Figure S14. Mass spectrum of [Fe(III)(L)$(Cl)_2$$(H_2O)_2$].
